# Moisturisers for the treatment of foot xerosis: a systematic review

**DOI:** 10.1186/s13047-017-0190-9

**Published:** 2017-02-07

**Authors:** Justin Parker, Rolf Scharfbillig, Sara Jones

**Affiliations:** Member, International Centre for Allied Health Evidence [iCAHE] and Sansom Institute University of South Australia, School of Health Sciences, University of South Australia, North Terrace, Adelaide, South Australia

**Keywords:** Dermatology therapy, Diabetes complications, Emollients

## Abstract

**Background:**

Xerosis, literally dryness of the skin, of the foot is a common condition encountered clinically, which can lead to discomfort and predisposition to infection. Currently, there are no evidence-based recommendations on which moisturiser formulations best alleviate xerotic symptoms. The aim of this review was to guide clinical practice in the treatment of primary and diabetes related foot xerosis, by identifying from the existing literature the most effective ingredient or formulation of topical treatments for symptoms of primary foot xerosis in the general population.

**Methods:**

A systematic review of published experimental trials was undertaken. Only studies pertaining to primary xerosis, classified within levels II – IV of the NHRMC hierarchy were reviewed. EMBASE, AMED, Cochrane, MEDLINE, CINAHL, Ageline and SCOPUS were searched using relevant search terms and keywords and pearling of reference lists was undertaken. Studies were evaluated for methodological quality using a critical appraisal tool. Individual active ingredients were identified from all studies, along with observed reported outcomes. A narrative synthesis was then conducted.

**Results:**

A total of 22 experimental studies were included, from which 12 different active ingredients were identified. Study literature consisted of mainly comparative studies against other active interventions or controls, or pre-post-tests and was of a poor-to-moderate methodological quality as assessed by the Epidemiological Appraisal Instrument. Urea was the most researched active ingredient (14 studies), with ammonium lactate being next (7 studies).

**Conclusions:**

No conclusive recommendations were possible due to wide variation in study quality, methodologies and outcome measures. A synthesis of available literature suggests that treatments containing urea as a primary active ingredient have been the most researched. The poor quality of literature generally, however, precludes recommendation of any active ingredient over another.

## Background

The term xerosis is used to describe dryness in the epidermal layers of the skin. It is a common condition, which can result in scaling, flaking and itching [1]. Risk factors for xerosis include sunlight, friction, low humidity, and use of soaps [2]. Xerosis also presents as a symptom of cutaneous conditions such as psoriasis, dermatitis and ichthyosis [3] with accompanying signs of inflammation and pain. The plantar area of the foot is particularly susceptible, due to its reliance on sweat secretions to remain hydrated [4].

It is important to adequately manage xerosis so that epidermal barrier function is maintained, serving to protect underlying tissues and structures from infection and physical damage [5]. Topical moisturisers are of benefit in managing xerosis [6], with many studies showing a demonstrable improvement in skin condition when comparing use of a moisturiser with a ‘sham’ base cream [7, 8].

Moisturising products achieve their hydrating and/or moisture barrier properties from active ingredients included in the formulation. These ingredients are broadly classed as occlusives, humectants, emollients or rejuvenators [5]. Categorised examples of some common active ingredients are shown in Table 1.

**Table 1:** Examples of common active ingredients in respective categories

Humectants	Occlusives	Emollients	Rejuvenators
Glycerin	Lanolin	Petrolatum	Collagen
Urea	Paraffin	Vegetable oil	Keratin
Ammonium lactate	Petrolatum	Dimethicone	Elastin
Gelatin	Cholesterol	Propylene glycol	
Hyaluronic acid	Stearyl alcohol	Castor oil	

Occlusives form a hydrophobic barrier over the skin, preventing trans-epidermal water loss. Their limitations include a ‘greasy’ feel, odour and possible inclusion of potential allergens [9]. Humectants enhance water transfer upward from the dermis to the epidermis and inwards from the external environment [9]. Emollients improve skin texture by filling gaps and fissures [9]. Rejuvenators are reported to act to replenish depleted essential skin proteins. These aid appearance by filling in fine lines [5].

An effective moisturiser will ideally include at least both an occlusive and humectant to achieve beneficial hydrating properties [10]. Although many products on the market include diverse combinations of these active ingredients [11], it appears no specific ingredient, formulation or product has been identified in the literature as optimal or superior for the treatment of plantar foot xerosis. One systematic review [12] that investigated treatments for dry skin found that moisturisers in general are effective, but no particular recommendations could be made due to lack of evidence. American guidelines on the treatment of atopic dermatitis state there are a lack of trials comparing moisturising agents in xerosis treatment (a prominent symptom of dermatitis), and the few that do exist do not display significant differences in efficacy [13].

The objective of this systematic review was to identify, collate and critically appraise relevant literature investigating efficacy of treatments for primary foot xerosis in a general and diabetic population. The aim was to identify any particular ingredient or formulation that gave superior results in treating primary xerosis symptoms in the current literature. The following specific review question was formulated as the focus of the review: What is the most effective ingredient or formulation of topical treatments for symptoms of primary foot xerosis in the general population?

## Methods

**Fig. 1:**
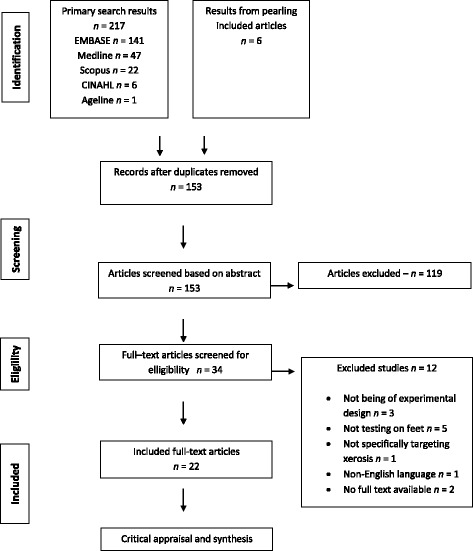
Preferred Reporting Items for Systematic Reviews and Meta – Analyses (PRISMA) diagram

A flowchart of the search strategy is included in Fig. 1. A systematic search was undertaken to identify literature relevant to the review question. A synthesis of the literature was then conducted to identify active ingredients tested and the treatment results. The goal was to identify any ingredient that had been evaluated across multiple studies for treating xerosis.

It was decided to include literature from all experimental study designs, as no well conducted randomised controlled trials were identified. Particular outcome measures could not be specified as a pre-requisite for inclusion, as the literature concerning the rating of xerosis severity varied markedly. There was an array of subjective and objective measurements described in the literature, and it was decided to include studies regardless of their specified outcome measures and allow them to be appraised using a rigorous critical appraisal tool. A narrative synthesis of included studies was then conducted to answer the clinical question.

It should be noted that while studies discussing xerosis as secondary to other diseases (such as eczema, psoriasis and ichthyosis) were excluded, studies focused on diabetic participants were allowed as the presentation and treatment of diabetic xerosis closely approximates that of xerosis in the non-diabetic population [6]. In both instances, the treatment goal is to increase and retain moisture and therefore, structural integrity [6]. Treatments for xerosis secondary to psoriasis or eczema often include corticosteroids and anti-inflammatory agents that counteract immunological responses in the skin, which are of little benefit to people outside of these populations [14].

Full exclusion and inclusion criteria for studies are included in Table 2.

**Table 2:** Inclusion and exclusion criteria for article search result

Inclusion	Exclusion
Experimental, quantitative study design Xerosis in foot/ft assessed by any method Studies after and including 1970 Primary xerosis or xerosis secondary to Diabetes mellitus only Published in English Human participants Full text articles	Xerosis in area other than foot/ft Xerosis secondary to disease (e.g. psoriasis, eczema)

### Registration

The systematic review was registered with the PROSPERO International prospective register of systematic reviews on 15/02/2015, registration no. CRD42015017032.

### Databases

**Fig. 2:**
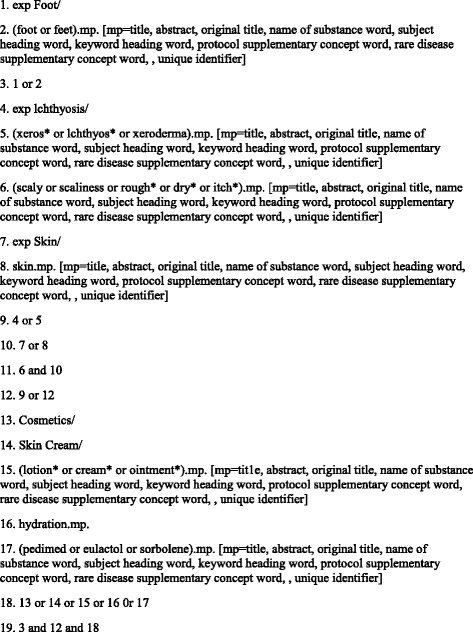
Medline search strategy

A PICO question (population, intervention, comparator, outcome) (Table 3) was devised to inform keywords, which were then used to search the following databases between September 20 and October 1 2014: EMBASE, AMED, Cochrane, MEDLINE, CINAHL, Ageline and SCOPUS. Search terms used were “xerosis” OR “dry skin” OR “ichthyosis” AND “feet” OR “foot” AND “moisturi*” OR “emollient” OR “humectant” OR “occlusive” OR “skin cream”. The following limits were applied when allowed: English language, years between 1970 – present, experimental trials, humans. A complete search strategy for Medline is shown in Fig. 2.

**Table 3:** PICO format clinical question

Population	General population and diabetics with foot xerosis
Intervention	Topical moisturisers
Comparator	Other moisturisers, placebo, no treatment
Outcome	Clinical scoring, instrumental measures

Intervention studies from Levels II through to IV of the National Health and Medical Research Council (NHMRC) Hierarchy of Evidence [15] were included. After duplicate references were removed, a title and abstract search was conducted by the principal investigator (JP). Articles that did not meet the eligibility criteria were then excluded. Included articles then underwent a ‘pearling’ process in which their reference lists were checked for articles missed in the initial search. All included articles were reviewed in full text by two independent assessors (JP and RS) against the Epidemiological Appraisal Instrument (EAI), a validated critical appraisal tool.

### Data extraction

Data were extracted to give an overview of the content of the included studies. Categories included were evidence level (as dictated by NHMRC) [15], population studied, interventions tested, sample size, study duration, outcome measures utilised, inclusion criteria and study results.

## Results

Thirty three articles qualified for full text review. Twelve were excluded for not meeting the eligibility criteria. Pearling through reference lists revealed 1 extra article, resulting in a total of 22 studies.

### Appraisal tool

Due to the varied nature of experimental designs in studies included in this review, it was necessary to find a critical appraisal tool that could be utilised across multiple experimental study designs. The Epidemiological Appraisal Instrument (EAI) developed by Genaidy et al. [16] was chosen as the validated and reliable methodological appraisal tool to determine risk of bias in these studies. Appraisal of methodological bias was performed using the EAI by two independent reviewers (JP and RS). Any discrepancies were discussed and resolved at a face-to-face meeting.

### Appraisal results

**Fig. 3:**
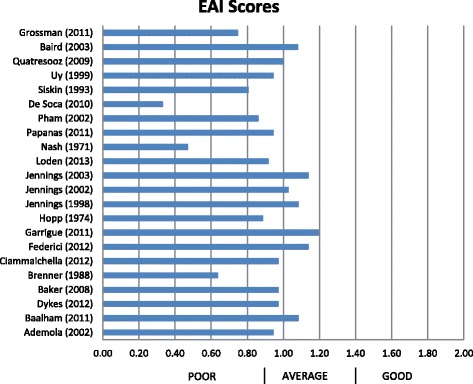
Epidemiological Appraisal Instrument (EAI) scores of study methodological quality

The results of the methodological appraisal have been condensed in Fig. 3. Of 22 studies, 15 scored in the ‘average’ category and 7 scored as ‘poor’.

### Populations

Nine [7, 8, 17–23] out of the 22 included studies featured samples with diabetes, with 2 of those solely focusing on Type 2 diabetes [17, 22]. Three studies were female–only [4, 19, 24] with one including only menopausal women [19]. Six studies had populations aged 40 years and over [1, 17, 21, 25–27]. The youngest reported age in any study was 13 [28] while the oldest was 97 years [25].

### Study designs

The included studies fell into levels III −2. III −3 or IV in the NHMRC hierarchy of evidence [15] for intervention studies, consisting of mainly comparative studies against other active interventions or controls, or pre-post-tests. Whilst several studies claimed to be randomised controlled trials, the descriptions indicated that these studies had no true controls or unsatisfactory randomisation of groups, and therefore could not be classified as level II evidence as defined in the NHMRC hierarchy. A large number of study designs featured participants testing different treatments on opposite feet, or having treatment applied to one foot while the other served as an untreated comparator. The least rigorous designs included were of a pre-post study design with no concurrent comparison groups (four studies).

Study durations ranged from 14 days to 7 months, with 28 days the most common duration (11 studies). Sample sizes ranged from 10 to 75 participants. Only one study [17] included a prospective sample size calculation to obtain estimates of cohort numbers required to detect clinically important differences between groups.

### Outcome measures

A range of outcome measurements were utilised throughout the literature. Primary outcome measures were either objective clinical scoring methods to rate xerosis symptom severity, or instrumental measurements to evaluate physiological characteristics, such as moisture loss. Outcomes measured clinically as indicators of skin dryness included flakiness, callosity, cracking, fissuring, scales, flexibility and tenderness. These were evaluated visually and by palpation, individually assigned a score and then summed together as part of a ‘composite’ scoring system. Examples of composite scoring systems were the XAS (xerosis assessment score) and Overall Dryness Severity Score [7, 8, 29]. A number of studies appeared to implement ad-hoc composite scoring methods, devised by individual study investigators to suit the needs of their experiment [18, 24, 28, 30].

Outcomes measured instrumentally as indicators of skin dryness included moisture retention, evaporation time, epidermal conductance, epidermal thickness, transcutaneous O_2_ and CO_2_ and skin pH.

No articles included in this review contained data pertaining to the validity or reliability (either inter or intra-rater) of the clinical or instrumental outcome measures utilised.

### Interventions

Table 4 lists the interventions used in each study. Most studies had interventions with different formulations of the tested products. All studies had some description of the most ‘active’ ingredients, and this review focused on these as being the most likely to contribute to any beneficial effect of the moisturiser. ‘Minor’ ingredient elements in each study have not been listed as it was beyond the scope of this study to investigate the individual physiological effects of these.

**Table 4:** Characteristics of included studies (listed in chronological order)

Study	NHMRC Level	Population	Sample Size (n male)	Intervention/active ingredients tested	Study duration	Outcome measurements	Inclusion Criteria	Study results	Mean values/Effect size
Nash [30]	IV	M/F, 12 – 87 years	75 (NR)	20% urea cream	7 months	Clinical scoring	UTD	Significant improvement after treatment	N/A
Hopp and Sundberg [27]	III-1	M/F, 60 + yrs	60 (NR)	Alpha-Keri (oil) vs Keri-lotion ® (both containing lanolin, mineral oil and emulsifiers) vs water soak vs control	12 days	Questionnaire, Dryness Scale, Panel evaluation	UTD	Water soak + Keri-lotion was superior to other combinations	Mean difference 1.16 (*P* < 0.05)
Brenner [25]	IV	M/F, 53 – 97 years	10 (7)	12% ammonium lactate	14 days	7-point Dry Skin Grading Scale	UTD	Significant improvement after treatment	N/A
Siskin et al. [29]	III-2	Sex NR, 24 – 85 years	55 (NR)	12% ammonium lactate vs no therapy	8 weeks	Overall Dryness Severity Score, Physician Global Improvement of Improvement/Worsening	≥ Moderate bilateral dryness	12% ammonium lactate superior to no treatment	Mean difference 0.98 (*P* < 0.05)
Jennings et al. [33]	III-3	M/F, 22 – 86 years	70 (34)	5% salicylic acid + 10% urea vs 12% ammonium lactate	28 days	Xerosis severity scale, Tenderness Scale, VAS	Mild-moderate bilateral xerosis	No significant difference between treatments	Mean difference 0.1 (*P* = 0.15)
Uy et al. [28]	III - 2	Sex NR, 13 – 72 years	57 (NR)	12% ammonium lactate vs liposome – based emollients (petrolatum, paraffin)	28 days	Clinical grading scores	≥ Moderate bilateral dryness and/or hyperkeratosis	No significant difference between treatments	N/A
Ademola et al. [2]	III-2	M/F, 18 – 65 years	25 (NR)	40% urea cream vs 12% ammonium lactate	28 days	Evapirometer (roughness), Corneometer (dryness), D-Squame (scales), VAS	≥ Grade 2 xerosis Free of cutaneous disease	40% urea cream superior	Effect size 0.19 (95% CI: −0.47 to 0.84)
Jennings et al. [36]	III-3	M/F, 26 – 83 years	35 (10)	10% lactic acid vs 12% ammonium lactate	28 days	Xerosis severity scale, Tenderness Scale, VAS	Mild-moderate bilateral xerosis	No significant difference between treatments, patients preference for 10% lactic acid	Mean difference 0.1 (*P* = 0.9)
Pham et al. [7]	III-2	M/F, age NR	40 (22)	10% urea + 4% lactic acid vs placebo vehicle	28 days	Xerosis Assessment Scale	≥18 years Type 1 or 2 diabetes Mild-moderate bilateral xerosis	10% urea + 4% lactic acid superior to placebo vehicle	N/A
Baird [20]	III-3	M/F, age NR	30 (14)	10% urea cream vs 25% urea cream	6 weeks	Customised equipment measuring skin electrical resistance	Type 1 or 2 diabetes Bilateral dry skin	25% urea cream superior to 10% urea cream	Effect size 0.27 (95% CI: −0.24 to 0.78)
Jennings et al. [37]	III-3	M/F, 18 + yrs	41 (NR)	Lanolin cream vs 12% ammonium lactate	28 days	Xerosis severity scale, Tenderness Scale, VAS	Moderate-severe bilateral xerosis	No significant difference between treatments	N/A
Baker and Rayman [21]	III-3	M/F, 40 – 74 years	26 (12)	10% urea foam vs ‘patient’s regular creams’ (aqueous cream, Diprotobase and Unguentum)	14 days	5-point scale for dryness, flexibility and callus formation	Type 1 or 2 diabetes Neuropathic Bilateral xerosis	10% urea foam superior to patient’s existing creams	Effect size −2.33) (95% CI: −2.99 to −1.59)
Quatrezoos et al. [19]	III-2	Female, 55 – 62 years	30 (0)	Chitlin – Glucan vs placebo vehicle + glycerol	35 days	Moisture Accumulation Test (MAT)	Menopausal women Type 1 or 2 diabetes Mod – severe xerosis	Chitlin-Glucan superior to placebo vehicle, equal result to glycerol yet longer-lasting	Mean difference of 60 points
De Soca and De Atencio [18]	IV	M/F, 20 – 50 year	40 (NR)	10% urea cream	28 days	Clinical scoring, VAS, Hydrometer, Skin pH	20 – 50 yo ‘Normal’ body weight Type 1 or 2 diabetes	Significant improvement after treatment	Mean difference of 5.4
Baalham et al. [4]	III-3	Female, age NR	15 (0)	Paraffin vs Paraffin + 10% urea	14 days	Digital moisture monitor	Adult Free of cutaneous disease Bilateral xerosis	Paraffin + 10% urea superior	Effect size 0.87 (95% CI: 0.1 to 1.59)
Garrigue et al. [8]	III-2	M/F, 18 – 75 years	54 (24)	Pedimed ® (urea, lactic acid, paraffin) vs placebo vehicle	28 days	Xerosis Assessment Score (XAS), D-Squame Corneometer	M / F 18 – 25 Type 1 or 2 diabetes Mod – severe xerosis	Pedimed ® superior to placebo vehicle	18% difference between groups (*P* < 0.05)
Grossman et al. [1]	IV	M/F, 41 – 70 year	12 (6)	35% urea foam	28 days	Clinical grading score, Global assessment score	≥18 years Xerosis diagnosis as per Global Assessment Score	Significant improvement after treatment	N/A
Papanas et al. [22]	III-2	M/F, age NR	20 (10)	10% urea foam vs no treatment	14 days	Corneometer	Type 2 diabetes	10% urea foam was superior to no treatment	Effect size 1.25 (95% CI: 0.55 to 1.9)
Ciammaichella et al. [23]	III-2	M/F, age NR	54 (29)	5% urea cream vs no treatment	28 days	Microangiopathy, Ultrasound, Partial O_2_ + CO_2_ pressures, VAS scale	Diabetes - Insulin treated Stable control Defined neuropathy	5% urea cream superior to no treatment	N/A
Dykes [24]	III-3	Female, 22 – 64 years	25 (0)	25% urea cream vs unspecified urea cream	14 days	Clinical photo scores, Corneometer	18+ years old Visibly dry feet Otherwise healthy	25% urea cream more effective than unspecified urea cream	Effect size −0.26 (95% CI: −0.83 to 0.35)
Federici, Federici and Milani [17]	III-2	M/F, 40 – 75 years	40 (16)	Urea, arginine and carnosine cream vs glycerol cream	28 days	Dryness Area Severity Index (DASI score), VAS score	40 – 75 years Mod – severe xerosis Type 2 diabetes	Urea, arginine and carnosine cream superior	Mean difference −0.8
Loden, von Scheele and Michelsen [3]	III-3	M/F, 21 – 86 years	50 (25)	15% alphahydroxy acid + 15% urea cream vs healthy controls	14 days	Trans-epidermal water loss (TEWL), Clinical scores, VAS	UTD	15% alphahydroxy acid + 15% urea significantly improved skin condition in both symptomatic and healthy samples	N/A

*III-2* comparative study with concurrent controls, *III-3* comparative study without concurrent controls, *IV* case series with either post-test or pre-test/post-test outcomes (as per [15]); *NR* not reported, *n* sample size, *yrs* years, *UTD* unable to determine, *n* sample size; *M* male; *F* female, *VAS* visual analogue scale; *CI* confidence interval

Twelve major active ingredients were identified in the literature. Urea was the most frequently listed primary active or co-active ingredient - it was tested in interventions across 14 of the included studies. This should not be construed as implying that urea is the most effective product, however, merely the most researched.

Alphahydroxy acid, arginine, carnosine and salicylic acid all appeared individually once in the literature. It should be noted that all were combined with urea, and hence it is likely that any beneficial effects observed cannot be solely attributed to them. Arginine and carnosine in particular were both combined with urea in comparison studies against a glycerol formulation. It is unclear what effect these ingredients may have when used individually.

### Follow up periods

Two studies [2, 28] featured a follow–up period, in which the longevity of beneficial effects was assessed after treatment stopped. Both studies found no statistical difference.

## Discussion

The aim of this systematic review was to identify the most effective ingredient or formulation of moisturiser to treat dry skin of the foot. The heterogeneous nature of populations, methodologies and outcome measures made meta-analysis of the literature impossible as a method of answering the review question.

Populations varied in the included literature, particularly in terms of age and diabetic status. One study [19] focussed on menopausal diabetic women, although it was not clarified how this group differed to other populations. Both increasing age and diabetes exacerbate the occurrence and severity of xerosis [26]. Including these alongside healthy younger populations in the review was deemed acceptable as the underlying pathophysiology and course of treatment for primary xerosis is similar regardless of age or diabetic status [6].

Out of the 22 studies included, only one [17] included a prospective sample size calculation. Without such a calculation, a study may have insufficient statistical power to be able to detect a clinically worthwhile difference between groups [31]. Accordingly, the results from the studies included in this review, even though some are statistically significant, may not reflect clinically worthwhile effects.

Outcome measures were particularly variable, including instrumental measurements, clinical scoring systems, ‘expert opinion’ and photographs. None of the studies reported validity or reliability testing of outcome measures or reporting of previous scores. This was curious for studies involving instrumental measures, as there is available literature validating several of these measures for epidermal hydration [32].

Numerous studies [3, 7, 17, 33] claimed to use a randomised trial design, but upon review were found to be randomising allocation of moisturisers to the left and right feet of participants, rather than randomising participants into distinct groups as dictated by NHMRC criteria [15]. As such, there were no true level II randomised controlled trials included in this review.

The overall quality of articles was ‘poor’ to ‘average’ when tested against a validated critical appraisal tool [16] with none appraised as ‘good’. Recruitment, randomisation and blinding techniques (if used) were often not explained in sufficient detail. Not all studies explained the criteria by which a diagnosis of ‘xerosis’ was made and how it was differentiated, for example, from a fungal infection or systemic disease.

Most studies in this review relied on the participant applying cream to their own feet. While self-application seems the most practical method for daily intervention application (especially when a study extends for weeks or months), there is no guarantee the participant will be compliant to the extent dictated by their respective trial, which may impact on efficacy data. Efforts were often made to control for this, by asking participants to fill out diaries and by weighing the contents of moisturiser bottles pre and post-trial. Application of cream by a blinded third party would appear the ideal method in these experimental study designs. The patient acceptance of the product will also have an impact on the compliance of the patient, should the product be greasy or difficult to apply, this may influence the patient applying the moisturiser regularly [34], and should be the subject of further studies.

Studies only mentioned major active ingredients. However, many treatments in these studies included an array of ‘minor’ ingredients. These included additives such as emulsifiers, alcohols and fats [35]. These additional ingredients may play a part in determining product efficacy and subsequently affect the outcomes of the studies.

As well as ingredient effectiveness, the question of ideal individual ingredient concentrations may also be raised. Two studies compared urea creams of different concentrations [20, 24], both showing that higher concentration urea creams had a superior effect. A 40% urea cream was shown to have a dramatically increased keratolytic effect on skin when compared to 10% urea cream [14]. Evaluating ideal ingredient concentrations to treat specific skin conditions would require the attention of more focussed studies.

It is not only desirable to identify the most effective moisturising ingredients, but also those with the longest–lasting effects after treatment cessation. Considering the ongoing nature of skin dryness and the challenge of patient compliance in treatment, it may be of clinical interest for a future paper to examine which ingredients or formulations produce the longest–lasting skin hydrating effects between applications.

Limitations of this review include the selection of articles by one author only and only full text and English language papers being included. A further limitation, due to the poor quality of the literature available with disparate outcome measures and minimal reporting of results, is that analysis methods such as effect size calculation could only be conducted on a small amount of the literature. Meta-analysis was unable to be conducted, thus limiting what could be construed with regard to most effective moisturiser from the review.

## Conclusion

A synthesis of available literature reveals that treatments containing urea as a primary active ingredient are the most prolifically researched for treating symptoms of xerosis in the foot. However, this observation is based on literature of a poor to average methodological quality. Larger-scale randomised trials comparing competing treatments would help ascertain optimum formulations and concentrations of ingredients for the treatment of foot xerosis. Furthermore, these trials should endeavour toward higher quality study designs in which they: (i) use validated and reliable outcome measures, (ii) conduct and report prospective power calculations for required sample numbers, (iii) treat individual participants as one sample, and (iv) have the intervention applied in a controlled environment to facilitate compliance.
